# Translation, cross-cultural adaptation, and psychometric validation of the Team Emergency Assessment Measure (TEAM) into European Spanish: a multi-perspective study including peer-assessment

**DOI:** 10.1186/s41077-026-00484-1

**Published:** 2026-09-09

**Authors:** Esther Leon-Castelao, Jose Luis Medina-Moya, Alejandro Durante-Lopez, Pedro Castro-Rebollo, Simon Cooper, Antoni Ruiz-Bueno, Jordi Bañeras

**Affiliations:** 1 Programa de Doctorat Medicina i Recerca Translacional, Facultat de Medicina i Ciències de la Salut, Universitat de Barcelona (UB), c. Casanova, 143, Barcelona, 08036 Spain; 2 Laboratori de Simulació Clínica, Facultat de Medicina i Ciències de la Salut, Universitat de Barcelona (UB), c. Casanova, 143, Planta Baixa, Barcelona, 08036 Spain; 3 Departament de Didàctica i Organització Educativa, Facultat d’Educació, Universitat de Barcelona (UB), pg. de la Vall d’Hebron, 171, Barcelona, 08035 Spain; 4 Servicio de Cardiología, Hospital Universitario 12 de Octubre, Av. de Córdoba, s/n, Madrid, 28041 Spain; 5 Medical Intensive Care Unit, Hospital Clínic de Barcelona, c. Villarroel, 170, Barcelona, 08036 Spain; 6 Facultat de Medicina i Ciències de la Salut, Universitat de Barcelona (UB), c. Casanova, 143, Barcelona, 08036 Spain; 7 Institut d’Investigacions Biomèdiques August Pi i Sunyer (IDIBAPS), c. Rosselló, 149, Barcelona, 08036 Spain; 8 School of Nursing and Midwifery, Edith Cowan University, 270 Joondalup Drive, Joondalup, WA 6027 Australia; 9 Departament de Mètodes d’Investigació i Diagnòstic en Educació, Facultat d’Educació, Universitat de Barcelona (UB), pg. de la Vall d’Hebron, 171, Barcelona, 08035 Spain; 10 Servei de Cardiologia, Hospital Universitari Vall d’Hebron, CIBERCV, Universitat Autònoma de Barcelona, pg. de la Vall d’Hebron, 119–129, Barcelona, 08035 Spain; 11 Vall d’Hebron Institut de Recerca (VHIR), SIMCES Research Group, Barcelona, Spain

**Keywords:** Teamwork, Non-technical skills, Simulation, Team emergency assessment measure, TEAM, Psychometric validation, Cross-cultural adaptation, Peer-assessment, European Spanish, High-fidelity simulation

## Abstract

**Background:**

The Team Emergency Assessment Measure (TEAM) is the most widely used tool for evaluating emergency team performance, with robust psychometric evidence across multiple languages and settings. However, no European Spanish version has been validated, and no previous TEAM study has simultaneously assessed teamwork from four complementary rater perspectives: instructors from the learners’ own specialty, instructors from other specialties, self-assessment by the performing participants, and peer-assessment by observing peers. This study aimed to translate, culturally adapt, and psychometrically validate a European Spanish version of the TEAM (s-TEAM) and to evaluate its reliability and validity across these four perspectives in emergency simulation scenarios, with peer-assessment representing a novel contribution of this work.

**Methods:**

The TEAM was translated and adapted into European Spanish following internationally recommended guidelines. The s-TEAM was applied across 216 high-fidelity cardiology emergency simulation scenarios within nine editions of a national training programme for second-year cardiology residents organised by the Spanish Society of Cardiology. Ratings were collected simultaneously from all four perspectives. Internal consistency, item analysis, concurrent validity, and factor structure (EFA and CFA on independent subsamples using polychoric correlations) were examined.

**Results:**

A total of 145 residents were registered across the nine course editions, with 30 participating instructors and 216 scenarios. The final analytical sample comprised 1,414 valid ratings. Internal consistency was excellent overall (Cronbach’s α = 0.921) and across all rater groups (range 0.901–0.938). The total score correlated strongly with the Global Rating Scale (*r* = .816). Factor analysis supported a predominantly unidimensional structure with a distinguishable Leadership/Communication facet (interfactor correlation 0.904–0.908). Peer-assessment yielded reliable scores (α = 0.901) comparable to other rater groups. Instructors from the residents’ own specialty (cardiologists) scored significantly lower than all other groups (d = − 0.42). Internal consistency was stable from the first scenario (α = 0.900) despite the absence of formal rater training.

**Conclusions:**

The s-TEAM is a valid, reliable, and feasible European Spanish adaptation of the TEAM. It produces consistent scores across four rater perspectives, enabling multi-source feedback during debriefing without additional faculty. These findings support its integration as both an assessment and formative tool within iterative simulation curricula.

**Supplementary Information:**

The online version contains supplementary material available at https://doi.org/10.1186/s41077-026-00484-1.

## Background

Communication failures and poor teamwork are consistently identified as major contributors to sentinel events, preventable harm, and mortality in increasingly complex healthcare systems [1–6]. The management of clinical emergencies therefore requires multidisciplinary teams that are aligned and coordinated [7–9]. Nevertheless, formal training in non-technical skills and team dynamics is not widely implemented [6, 9–11], despite strong evidence that such training improves team performance, reduces errors, and improves patient outcomes [12, 13].

Measuring teamwork performance in educational settings allows educators to identify gaps and provide targeted feedback, thereby enhancing the effectiveness of training interventions [14, 15]. Simulation-based education is particularly well suited to this purpose, as it offers a controlled and reproducible environment in which multiple simultaneous observers can rate the same team performance [16]. Several instruments have been developed to measure teamwork in emergency contexts [17–19], among which the Team Emergency Assessment Measure (TEAM), originally designed by Cooper and colleagues [20, 21], is the most widely used and has consistently demonstrated strong psychometric properties across multiple language adaptations and settings [22–24]. However, two gaps remain. First, no European Spanish version of the TEAM has been validated to date. Second, the TEAM was developed for use by trained expert observers [20, 21] and has subsequently been shown to be reliable in the hands of expert clinicians without instrument-specific training and of novice observers after rater training [23, 24]. Whether the participants in a simulation themselves, as self-assessors of their own performance and as peer-assessors of the teams they observe, can produce reliable ratings without formal training has not been examined, and no previous TEAM study has collected four concurrent rater perspectives within the same programme.

The present study therefore had two aims: first, to develop a European Spanish adaptation of the TEAM (s-TEAM) through translation and cross-cultural adaptation; and second, to evaluate its reliability and validity when applied concurrently from four complementary rater perspectives (instructor-cardiologists, instructor non-cardiologists, self-assessment by performing residents, and peer-assessment by observing residents) within a national simulation-based cardiology training programme. We hypothesised that resident-generated ratings — both self- and peer-assessment — would be internally consistent and would converge with instructor ratings; that reliable measurement would be attainable after brief familiarisation, without formal rater training; and that team performance scores would improve across the course scenarios.

## Methods

### Instrument description

The Team Emergency Assessment Measure (TEAM) [20, 21] is a validated instrument developed to assess non-technical skills in emergency teams, focusing on leadership, communication, cooperation, coordination, team climate, adaptability, situation awareness, prioritisation, and adherence to clinical standards. It comprises 11 items, originally conceptualised across three domains: leadership (2 items), teamwork (7 items), and task management (2 items) rated on a five-point Likert scale from 0 (Never) to 4 (Always), with a total score ranging from 0 to 44. In addition, a Global Rating Scale (1–10) provides an overall judgement of team performance, conceived as a holistic “gut reaction” [20, 21, 23]. Validation studies in several languages and contexts have consistently reported strong internal consistency and robust psychometric properties [22–24].

### Study design

This was a translation, cross-cultural adaptation, and psychometric validation study of the Team Emergency Assessment Measure (TEAM) into European Spanish, conducted in a simulation-based educational setting and following international recommendations for cross-cultural adaptation [25]. The final European Spanish version (s-TEAM) was applied over a 6-month period in 216 cardiology emergency simulation scenarios, which formed the basis for the validation study. The study was coordinated by the Spanish Society of Cardiology (Sociedad Española de Cardiología) and conducted at IAVANTE, a regional simulation centre in Andalusia, Spain. The simulation setting made it possible to collect concurrent ratings from all four rater perspectives within the same scenario, a design that would be impractical during real clinical emergencies.

### Instrument translation and cross-cultural adaptation

**Fig. 1:**
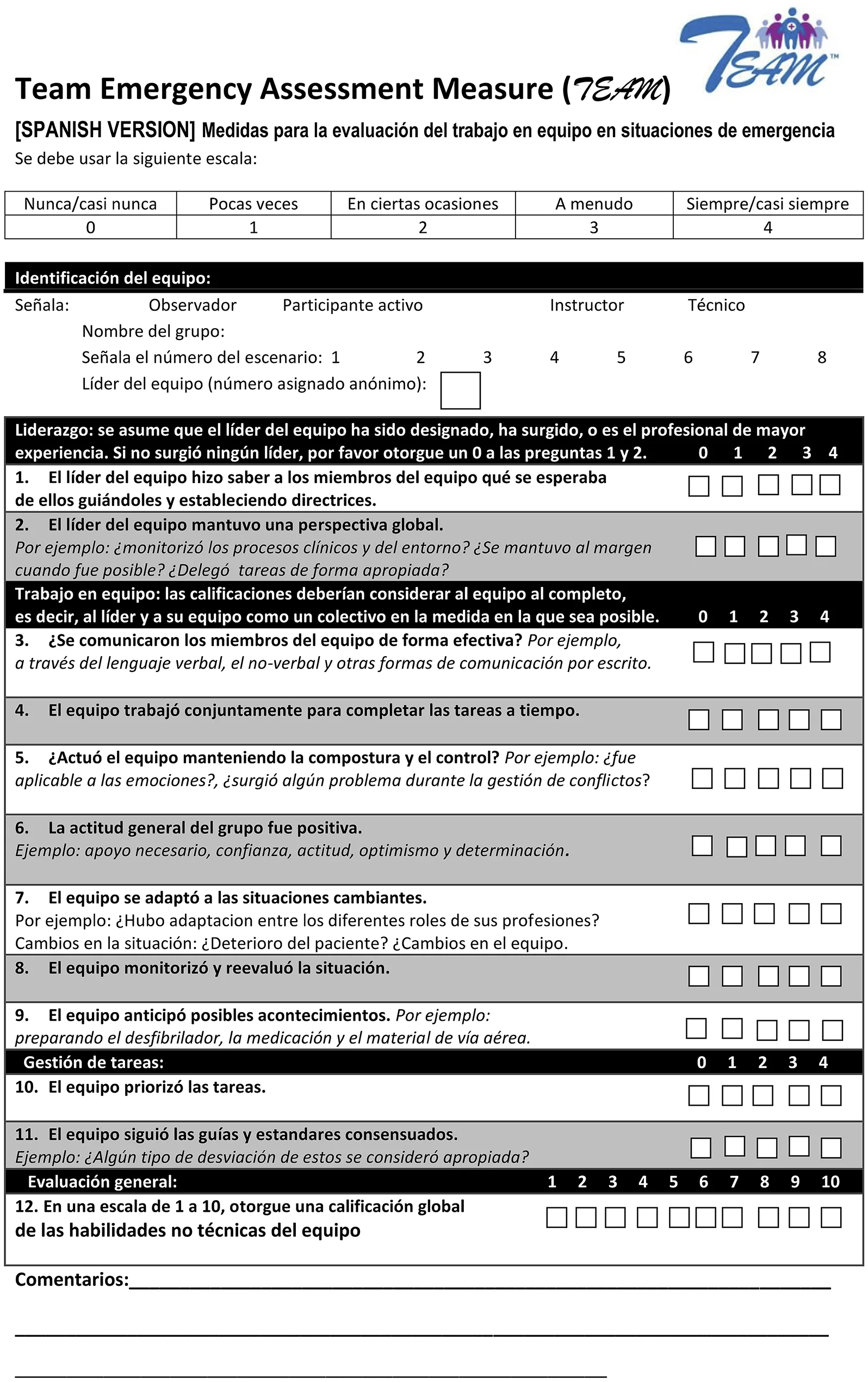
Team emergency assessment measure, European Spanish version (s-TEAM)

After obtaining permission from the original author, the TEAM European Spanish adaptation (s-TEAM) followed internationally recommended steps [25]: (1) independent forward translations by seven translators: two physicians and three nurses, all native Spanish speakers with expertise in critical care, simulation-based education, and the assessment of non-technical skills (one of them with prior experience using the original TEAM in English-speaking settings), plus a professional scientific translator (a native English speaker) and a professional medical translator (a native Spanish speaker); (2) synthesis of a consensus version by three bilingual members of the translation group, with cultural adaptation of the language to the European Spanish context and consultation with the original author to clarify the meaning of grammatical and lexical constructions in which doubts or discrepancies arose; (3) independent back-translations by two professional translators (one a native English speaker) who had no knowledge of the original instrument; and (4) harmonisation, in which the consensus group and the original TEAM author reviewed the reconciled back-translated version, resolved a wording discrepancy concerning the instrument’s title, and confirmed semantic and conceptual equivalence with the original. The final European Spanish version incorporated this recommendation and was designated s-TEAM (Fig. 1).

### Participants and eligibility criteria

Participants were second-year cardiology residents enrolled in the national simulation training programme organised by the Spanish Society of Cardiology (SEC). All residents attending the course during the study period were eligible. Data collection was fully anonymous; no demographic data were recorded. Inclusion criteria: All completed s-TEAM forms with rater role recorded were eligible for analysis. Exclusion criteria: Forms were excluded if: (a) they were illegible or entirely blank; (b) for psychometric analyses requiring complete item-level data (Q1–11), any item response was missing or contained an out-of-range value (i.e., outside the 0–4 response scale). Forms without scenario identification (*n* = 42) were retained in the analytical sample but excluded from scenario-level analyses. No imputation was performed; listwise deletion was applied.

### Protocol and data collection

Data were collected within the national two-day educational programme organised by the Spanish Society of Cardiology (SEC) and delivered to all second-year cardiology residents in Spain [26, 27]. During the study period, a total of 9 courses were conducted, each following the same standardised structure. Key elements of the simulation design, equipment, participant orientation, faculty roles, and instructional format are summarised in Table 1, following the reporting recommendations by Cheng et al. [28].

Each course spanned two days and included eight high-fidelity cardiology emergency simulation scenarios (six on Day 1, two on Day 2), alongside technical workshops not included in this study. Residents were divided into subgroups and each resident was scheduled to participate in eight simulation scenarios: four as an active participant and four as an observer. Each scenario session lasted approximately one hour (10-minute briefing, 10-minute simulation, 40-minute debriefing) and was facilitated by two instructors (one cardiologist and one non-cardiologist) supported by a confederate nurse.

**Fig. 2:**
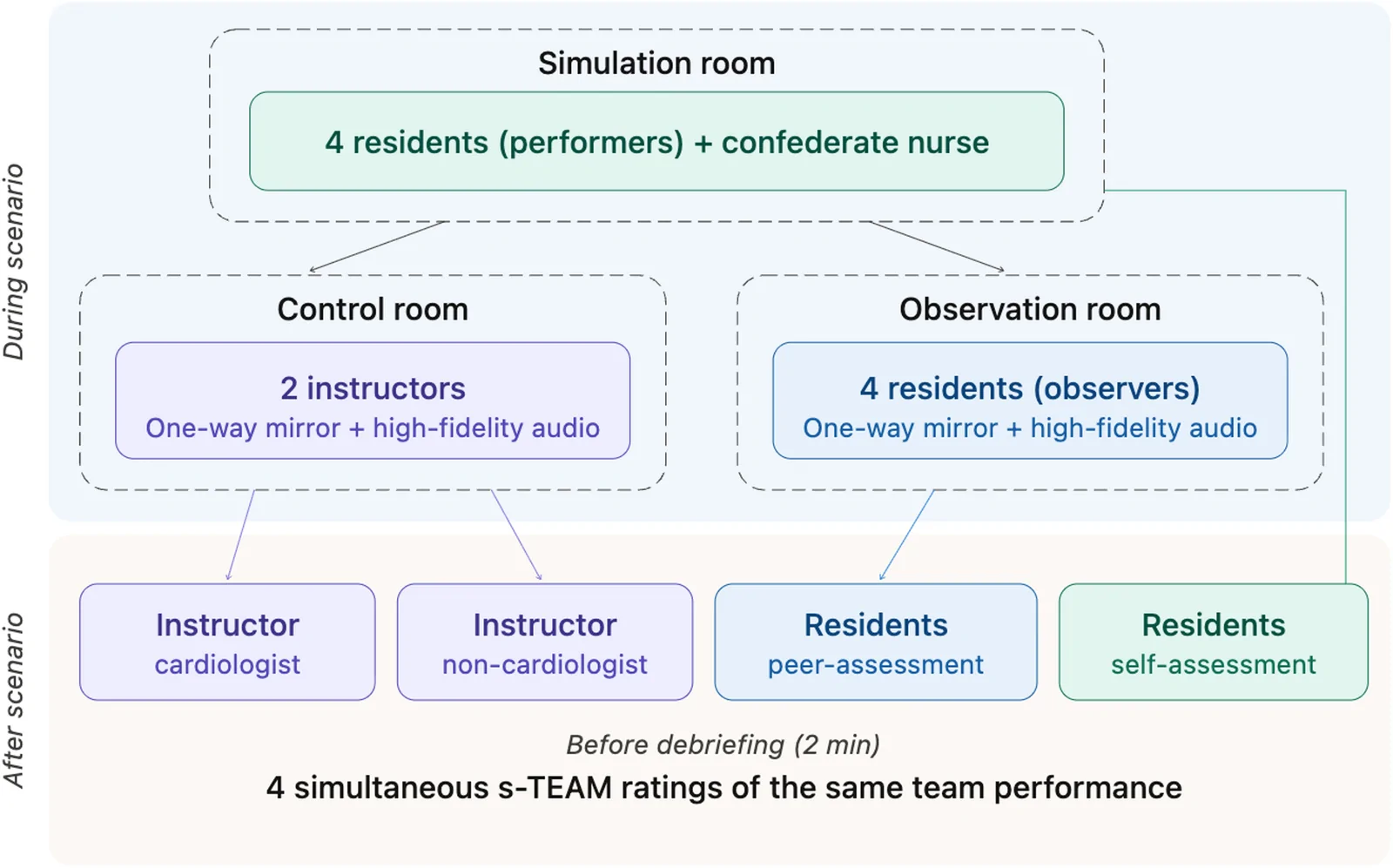
Data collection process and rater perspectives. Note: Schematic representation of the data collection protocol. During each simulation scenario, four concurrent rating perspectives were obtained: instructor-cardiologist (from control room), instructor non-cardiologist (from control room), peer-assessment by observing residents (from observation room), and self-assessment by performing residents (from simulation room). All ratings were completed immediately after each scenario and before the debriefing session. Nine course editions were conducted, each comprising three teams and eight scenarios, yielding 216 team-scenario observations

Scenarios were run from a control room with direct view through a one-way mirror and high-fidelity audio, while observers were located in a separate room with one-way mirror and high-fidelity audio. A 30-minute prebriefing at the start of each course established a safe and structured learning environment, and each scenario was followed by a 40-minute debriefing led by the cardiologist instructor, structured according to the Promoting Excellence and Reflective Learning in Simulation (PEARLS) framework [29]. To avoid bias from the debriefing discussion, all ratings were completed immediately after each scenario and before the debriefing (Fig. 2).

Raters were given two minutes to complete a printed s-TEAM form. Each form was coded with date, scenario number (1–8), and rater perspective. Completed forms were collected by the course coordinator and subsequently entered into a centralised database.

**Table 1:** Simulation design characteristics following reporting elements for healthcare simulation research

Element	Subelement	Description
*Participant orientation*	*Orientation to the simulator*	30-minute prebriefing at the start of each course. Participants were introduced to the simulators (Laerdal SimMan 3G and CAE HPS), their capabilities, and how to interact with them in the simulated environment
	*Orientation to the environment*	During the same prebriefing, participants were oriented to the simulation rooms, control room, observation room (one-way mirrors and high-fidelity audio), emergency equipment layout, and the learning objectives for the session
*Simulator type*	*Simulator make and model*	(1) Laerdal SimMan 3G (Laerdal Medical, Stavanger, Norway); (2) CAE Human Patient Simulator (HPS; CAE Healthcare, Sarasota, FL, USA)
	*Simulator functionality*	The SimMan 3G provided programmable cardiac rhythms (including 12-lead ECG), haemodynamic parameters, respiratory function, palpable pulses synchronised with ECG, and real defibrillation capability (LiveShock). The CAE HPS additionally featured autonomous physiological modelling with true oxygen and CO₂ gas exchange, the ability to support mechanical ventilation, and automated pharmacological responses. No modifications were made to either simulator
*Simulation environment*	*Location*	IAVANTE, Centro de Simulación Clínica Avanzada, Fundación Pública Andaluza Progreso y Salud, Parque Tecnológico de Ciencias de la Salud, Granada, Spain (dedicated simulation centre)
	*Equipment*	Three concurrent fully equipped simulated emergency rooms, each containing bedside monitoring, a defibrillator, an emergency drug cart, and standard resuscitation equipment (airway management devices, intravenous access, medications). Control room with direct view through one-way mirror and high-fidelity audio. Separate observation room with one-way mirror and high-fidelity audio for peer-assessment
	*External stimuli*	No additional external stimuli were introduced
*Simulation event/scenario*	*Event description*	Eight standardised, scripted cardiology emergency scenarios with pre-programmed physiological progressions and triggers. Scenarios had been refined over successive course editions prior to the study
	*Learning objectives*	Non-technical skills (leadership, communication, teamwork, situation awareness) and clinical decision-making in time-critical cardiac emergencies
	*Group vs. individual practice*	Group practice. Residents divided into six subgroups per course (3–4 residents per subgroup). In each session, three subgroups performed as active teams in three concurrent scenarios while three subgroups observed. The structured rotation was designed to ensure that each resident acted in four scenarios and observed four
	*Use of adjuncts*	No moulage was used. Standard clinical props (IV lines, syringes, medications) were available
	*Facilitator/operator characteristics*	Two instructors per scenario: one cardiologist and one non-cardiologist specialist. Instructors underwent a structured training programme in simulation-based education and debriefing in 2018 (one-day in-person course + online follow-up), delivered by a simulation expert from the SEC. Familiar with concepts of team performance; had not used the TEAM instrument before the study (brief pre-course familiarisation only; see Raters)
	*Pilot testing*	Scenarios had been piloted and iteratively refined across multiple course editions prior to the study period
	*Confederates/standardised patients*	One confederate nurse per scenario, following standardised instructions to ensure consistency across the nine course editions. Confederate was a simulation centre staff member experienced in high-fidelity simulation facilitation
*Instructional design*	*Duration*	Two-day course. Day 1: six simulation sessions + catheter workshop. Day 2: two simulation sessions + intubation workshop. Each session ≈ 1 h (10-min briefing, 10-min simulation, 40-min debriefing). Total simulation exposure: ≈8 h per resident across eight scenarios
	*Timing*	s-TEAM ratings completed immediately after each simulation and before the debriefing session (within 2 min)
	*Frequency/repetitions*	Eight scenarios per resident (four as performer, four as observer). No scenario was repeated for the same resident
	*Clinical variation*	Eight distinct scenarios: ST-elevation myocardial infarction, cardiac arrest, acute arrhythmias, and cardiogenic shock
	*Standards/assessment*	No predefined pass/fail standards. The s-TEAM was used as a formative assessment tool; scores were not disclosed to residents during the course
	*Adaptability of intervention*	Scenario progression was standardised and not adapted to individual learner performance
	*Range of difficulty*	Scenarios designed to present varying levels of clinical complexity
	*Non-simulation interventions*	Day 1: catheter workshop. Day 2: intubation workshop. These technical workshops were not included in the study
	*Integration*	The programme is part of the SEC’s established national simulation curriculum for second-year cardiology residents, delivered annually across Spain
*Feedback and debriefing*	*Source*	Cardiologist instructor (facilitator-led)
	*Duration*	40 min per scenario
	*Facilitator presence*	Yes. The cardiologist instructor led the debriefing; the non-cardiologist co-instructor was present
	*Facilitator characteristics*	As described under Facilitator/operator characteristics above. Additionally trained in the PEARLS debriefing framework
	*Content*	Teamwork processes (leadership, communication, coordination) and clinical decision-making
	*Structure/method*	PEARLS framework (Promoting Excellence and Reflective Learning in Simulation): learner self-assessment phase, focused facilitation phase, and directive feedback/teaching phase
	*Timing*	Terminal debriefing immediately after each scenario. s-TEAM ratings collected before debriefing
	*Video*	Not used during debriefing
	*Scripting*	No debriefing script. Instructors followed the PEARLS framework structure

Note. Reporting elements follow the extensions to the CONSORT and STROBE statements for healthcare simulation research (Cheng A, Kessler D, Mackinnon R, et al. Adv Simul. 2016;1:25). SEC = Sociedad Española de Cardiología (Spanish Society of Cardiology); HPS = Human Patient Simulator; PEARLS = Promoting Excellence and Reflective Learning in Simulation; s-TEAM = European Spanish version of the Team Emergency Assessment Measure

### Raters

The validation was structured around four rater perspectives: instructor-cardiologists, instructor non-cardiologists, self-assessment, and peer-assessment. In each scenario, ratings were provided by the two instructors (one cardiologist and one non-cardiologist specialist), by the performing residents (self-assessment), and by the observing residents (peer-assessment); the course design ensured that each resident contributed data from both resident perspectives, acting in four scenarios and observing in four. The three confederate nurses took part inside the scenarios and were not included as raters.

The nine course editions were delivered by 30 instructors: 15 cardiologists, who led the scenarios and debriefings, and 15 non-cardiologist specialist co-instructors — anaesthesiology (*n* = 5), angiology and vascular surgery (*n* = 3), emergency medicine (*n* = 2), family and community medicine (*n* = 4), and intensive care medicine (*n* = 1) — all of whom work in areas related to emergency or critical care. All instructors had expertise in simulation-based education, were trained in structured debriefing techniques, and were familiar with concepts of team performance from previous training courses, but none had used the TEAM instrument before the study. Residents were second-year cardiology trainees without prior training in educational assessment or structured teamwork evaluation and had not previously used TEAM; the course did not include a dedicated theoretical session on non-technical skills, so residents relied on the heterogeneous knowledge acquired during their residency training, with non-technical aspects addressed through feedback during the debriefings.

Instrument familiarisation was deliberately brief and differed by role. Instructors attended a pre-course familiarisation session covering the simulation centre and the educational programme, which included an approximately 10-minute explanation of the s-TEAM and of the requirement to complete it after each scenario; subsequently, on each course day, the course coordinator reminded instructors of the s-TEAM procedure during the faculty prebriefing. Residents received an approximately 5-minute explanation during the 30-minute prebriefing, in which the form was shown and the three dimensions and each item were read; after each scenario, printed forms were handed out before the debriefing, with a reminder to complete them and the opportunity to ask questions. No formal rater training, practice ratings, or calibration exercises were provided to either group. For exploratory purposes, instructors were analysed separately (cardiologists vs. non-cardiologists). Because data collection was fully anonymous and no individual rater identifiers were recorded, ratings could not be linked across scenarios to specific evaluators; consequently, formal inter-rater reliability coefficients at the individual rater level (e.g., ICC across identifiable raters) could not be estimated.

### Statistical analysis

Data were analysed using IBM SPSS Statistics (version 30) and FACTOR 12.06.07 [30]. Descriptive statistics were calculated for each of the four rater groups: instructor-cardiologists, instructor non-cardiologists, residents peer-assessment (observers), and residents self-assessment (performers).

Given the ordinal nature of the five-point response scale and the potential for non-normal item distributions, polychoric correlations were used for all factor analyses. Parametric tests (ANOVA, Pearson correlations) were employed as primary analyses, supported by their robustness with large samples; non-parametric tests (Kruskal–Wallis, Mann–Whitney U, Spearman’s ρ, Jonckheere–Terpstra) were conducted as sensitivity analyses throughout.

#### Internal consistency and item analysis

Cronbach’s α was computed for the 11-item scale and for each domain (Leadership, Teamwork, Task Management), both overall and stratified by rater role. The 95% confidence intervals for α were calculated using Feldt’s F-based method [31], and α values were interpreted according to conventional benchmarks [32]. Internal consistency was examined primarily for the 11 analytical items (Q1–11). Alpha values including the GRS were also reported for comparability with previous TEAM studies, which have computed α either for the 11 analytical items alone [20] or for the full 12-item instrument including the GRS (Table 6). Corrected item–total correlations and α-if-item-deleted values were examined to assess each item’s contribution to the construct, with a minimum threshold of 0.30 for item retention. Mean and range of inter-item correlations were also computed. Floor and ceiling effects were assessed as the percentage of ratings at the minimum (0) and maximum (4) scale points, with values exceeding 15% considered notable [33].

#### Concurrent validity

Pearson correlations of each item, domain scores, and the 11-item total score with the GRS (item 12) were computed using pairwise deletion due to 17 missing GRS values (*n* = 1,397).

#### Construct validity

The dimensionality of the s-TEAM was examined through exploratory factor analysis (EFA) followed by confirmatory factor analysis (CFA). Polychoric correlations were used throughout, given the ordinal nature of the response scale [34]. The total analytical sample (*N* = 1,414) was split into two independent subsamples using the Solomon method [35]: Sample 1 (*n* = 729) for EFA and Subsample 2 (*n* = 666) for CFA; 19 observations were excluded during the splitting optimisation process (Ratio Communality Index = 0.992). The number of factors was determined through optimal parallel analysis based on minimum rank factor analysis [36]. EFA employed Robust Unweighted Least Squares (RULS) extraction with Robust Promin oblique rotation; CFA used the Mild-Restricted approach [37] with LOSEFER-corrected chi-square statistics [38]. Model fit was evaluated using CFI, NNFI (Tucker–Lewis Index), RMSEA with bootstrap confidence intervals, and χ²/df. Both one-factor and two-factor solutions were explored via EFA on Sample 1. The two-factor structure was subsequently confirmed via CFA on the independent Subsample 2. For descriptive purposes, a supplementary one-factor CFA was also examined in Sample 1 to further characterise the fit of the unidimensional solution identified in the exploratory analyses. 

#### Comparisons across rater roles

Differences across the four rater groups were examined using one-way ANOVA with Welch’s F where Levene’s test indicated unequal variances, and Games-Howell post-hoc comparisons. Effect size for the overall ANOVA was estimated using partial eta-squared (η²p); Cohen’s d was computed for planned pairwise comparisons. Non-parametric tests (Kruskal–Wallis, Mann–Whitney U) were conducted as sensitivity analyses. 

#### Progression across scenarios

The trend in total scores across the eight scenarios was assessed using ANOVA with a polynomial linear contrast and Pearson’s r. Internal consistency was examined per scenario. Non-parametric sensitivity analyses included Spearman’s ρ and the Jonckheere–Terpstra test.

The significance level was set at *p* < .05 for all analyses. Listwise deletion was applied for all analyses involving the 11-item scale (Q1–11); pairwise deletion was used for correlations involving item 12 (GRS).

### Use of large language models

A large language model (Claude, Anthropic) was used as a writing aid to improve the readability and English language quality of the manuscript, and to assist in the generation of the data collection flowchart (Fig. 2). All outputs were critically reviewed, verified against the original data, and revised by the authors, who retain full responsibility for the content.

### Ethics approval and consent to participate

The study protocol was approved by the Research Ethics Committee of IAVANTE. All procedures were conducted in accordance with the Declaration of Helsinki. Participation was voluntary and data collection was fully anonymous; completed forms were not linked to any identifiable participant information. Informed consent was obtained from all participants prior to data collection. No financial compensation or academic incentives were offered.

## Results

### Cross-cultural adaptation

The forward–backward translation process resulted in minor linguistic adjustments to ensure conceptual equivalence in the European Spanish context. No substantive discrepancies were identified in the content of the 11 analytical items or their rating prompts. The back-translation revealed one terminological discrepancy regarding the instrument’s title, specifically, whether the English term should be rendered as a reference to a tool or to a measurement, which was resolved through consultation with the original TEAM author (S. Cooper). The 12-item structure was fully retained (11 analytical items plus one Global Rating Scale), and the final European Spanish version was designated s-TEAM (Fig. 1).

### Participants

A total of 1,541 s-TEAM forms were collected across nine course editions involving 145 registered second-year cardiology residents (12–18 per course) and 216 team-scenario observations. Because the anonymous design did not register scenario-level attendance or team composition, the exact number of forms that could have been completed cannot be reconstructed; the 1,541 collected forms correspond to an average of 7.1 forms per scenario, of a possible eight to ten (two instructors, three to four performing residents, and three to four observing residents), indicating an overall completion rate of approximately 71–89%. Of the collected forms, 43 were excluded prior to data entry due to illegibility or entirely blank responses, yielding 1,498 digitised records. For psychometric analyses requiring complete item-level data (Q1–11), 82 forms with missing responses and 2 with out-of-range values were excluded, yielding a final analytical sample of 1,414 ratings: instructor-cardiologists (*n* = 192), instructor non-cardiologists (*n* = 158), residents as peer-assessors (*n* = 522), and residents as self-assessors (*n* = 542). No imputation was performed; listwise deletion was applied. Of the 1,414 ratings, 1,372 (97.0%) had a recorded scenario number, and the remaining 42 were excluded from scenario-level analyses (see Additional file 1 for the exclusion flowchart).

### Reliability

Internal consistency was excellent for the total sample (Cronbach’s α = 0.921 [95% CI 0.915, 0.927] for the 11-item scale; α = 0.929 [0.923, 0.934] for the 12-item version including the GRS) (Table 2). Subgroup analyses by rater role yielded consistently high values (α range 0.901–0.938 for the 11-item scale), with no rater group falling below the 0.90 threshold. At the subscale level, Leadership and Teamwork showed excellent reliability across all groups (α ≥ 0.856), while Task Management yielded adequate to good values (α = 0.862–0.918). Full results stratified by rater role and domain are reported in Table 2.

**Table 2:** Internal consistency (Cronbach’s α [95% CI]) of the s-TEAM by rater role

Rater group	*n*	Full scale		Subscales (11-item)		
		12-item (11 items + GRS)	11-item	Leadership	Teamwork	Task Mgmt
**All raters**	1414	0.929 [0.923, 0.934]	0.921 [0.915, 0.927]	0.902 [0.891, 0.912]	0.895 [0.886, 0.903]	0.862
Instr.-cardiologists	192	0.941 [0.928, 0.953]	0.935 [0.920, 0.948]	0.884 [0.846, 0.913]	0.904 [0.882, 0.923]	0.918
Instr. non-cardiologists	158	0.942 [0.928, 0.955]	0.938 [0.923, 0.951]	0.856 [0.803, 0.895]	0.918 [0.897, 0.936]	0.880
Peer-assessment	522	0.912 [0.900, 0.923]	0.901 [0.888, 0.913]	0.917 [0.901, 0.930]	0.862 [0.843, 0.879]	0.862
Self-assessment	542	0.927 [0.918, 0.936]	0.918 [0.907, 0.928]	0.904 [0.886, 0.919]	0.897 [0.883, 0.910]	0.876

Note. GRS = Global Rating Scale (item 12, rated 1–10). The 12-item version comprises the 11 analytical items (Q1–Q11) plus the GRS; the 11-item version (Q1–Q11, rated 0–4) constitutes the core summative scale (range 0–44). Leadership = items 1–2; Teamwork = items 3–9; Task Management = items 10–11. 95% CI computed using Feldt’s (1965) F-based method. The n for the 12-item scale is lower (All = 1397; INC = 157; Peer = 514; Self = 534) due to missing Q12 (GRS) values. Listwise deletion applied

### Descriptive statistics and item analysis

Item-level and scale-level analysis are presented in Table 3. Floor effects were negligible (≤ 3.6%), while ceiling effects exceeded 15% on all items, reaching 55.9% for Q6 (morale). The total score averaged 34.37 (SD = 6.92; 78.1% of maximum), with Leadership rated proportionally lower (72.4%) than Teamwork (79.9%) and Task Management (77.9%). All corrected item–total correlations exceeded the 0.30 retention threshold (range 0.596–0.757), and no item deletion improved the overall α (0.921) (Table 3).

### Concurrent validity

The Global Rating Scale (item 12; M = 8.02, SD = 1.39) was strongly correlated with the 11-item total score (*r* = .816, *p* < .001), supporting the concurrent validity of the s-TEAM. Individual item–GRS correlations ranged from 0.534 (Q1) to 0.683 (Q10), and domain–GRS correlations were 0.581 for Leadership, 0.783 for Teamwork, and 0.741 for Task Management (all *p* < .001) (Table 3).

**Table 3:** Descriptive statistics, item analysis, and concurrent validity (*N* = 1414)

Item	M	SD	Floor (%)	Ceiling (%)	Item–total *r*	α if del.	Item–GRS *r*
*Leadership*							
1. Leadership direction	2.85	0.99	3.6	25.7	0.596	0.919	0.534
2. Global perspective	2.94	0.99	3.2	31.3	0.654	0.916	0.574
*Teamwork*							
3. Effective communication	3.24	0.77	0.1	41.7	0.656	0.915	0.568
4. Worked together timely	3.26	0.76	0.2	42.7	0.708	0.913	0.606
5. Composure and control	3.24	0.81	0.1	43.8	0.698	0.913	0.634
6. Positive morale	3.45	0.71	0.1	55.9	0.687	0.914	0.576
7. Adapted to changes	3.12	0.85	0.6	36.9	0.714	0.912	0.646
8. Monitored and reassessed	3.18	0.83	0.4	40.5	0.694	0.913	0.601
9. Anticipated potential actions	2.87	0.92	1.2	26.3	0.717	0.912	0.660
*Task Management*							
10. Prioritised tasks	3.14	0.78	0.5	34.4	0.757	0.910	0.683
11. Followed standards	3.08	0.82	0.6	32.8	0.726	0.912	0.673
**Total score (Q1–Q11)**	**34.37**	**6.92**				**0.921**	**0.816**
12. Global Rating Scale (1–10)	8.02	1.39					—

Note. Items Q1–Q11 rated 0 (never) to 4 (always); Q12 rated 1–10. Floor/Ceiling = % at minimum (0) or maximum (4). Item–total r = corrected item–total correlation; values > 0.30 indicate acceptable contribution. α if del. = Cronbach’s α if item removed; values ≤ 0.921 confirm retention. Item–GRS r = Pearson correlation with Global Rating Scale. All correlations *p* < .001. Item–GRS correlations computed pairwise (*n* = 1397). Listwise deletion applied for the 11-item scale

### Factor structure

Sampling adequacy was very good (KMO = 0.927 [95% CI 0.903, 0.930]; Bartlett’s χ² = 6,842.2, df = 55, *p* < .001). Two eigenvalues exceeded unity (7.12 and 1.07), accounting for 64.8% and 9.7% of the total variance, respectively; however, optimal parallel analysis based on polychoric correlations [36] recommended retaining a single factor, as only the first eigenvalue exceeded the 95th percentile of the random data distribution. The one-factor EFA on Sample 1 (*n* = 729) yielded excellent model fit after LOSEFER correction (RMSEA = 0.012 [95% CI 0.012, 0.013]; CFI = 0.999; NNFI = 1.000; LOSEFER-corrected χ²(44) = 48.70, *p* = .289). All 11 items loaded substantially on the single factor (range 0.633–0.858; Table 4), with factor score reliability of 0.950 (EAP). A supplementary Mild-Restricted CFA [37] on the same sample confirmed these findings, with all fit indices within the excellent range (Table 4).

Inspection of the standardised residuals from the one-factor model revealed a consistent pattern of local dependence aligned with the original TEAM conceptual domains [20]. The two Leadership items (Q1–Q2) exhibited markedly elevated residuals (EFA: 12.00; CFA: 13.69), reflecting a polychoric inter-item correlation (*r* = .891) beyond what the single factor accounts for and consistent with their comparatively lower factor loadings (0.633 and 0.705 in EFA; 0.582 and 0.659 in CFA). Smaller residuals were observed between Q5–Q6 (2.82/3.09), Q3–Q4 (2.58/2.78), and Q10–Q11 (2.95 in CFA). This pattern, together with the theoretical three-domain structure of the TEAM, motivated the exploration of a two-factor model.

EFA with Robust Promin oblique rotation on Sample 1 yielded two factors: Factor 1 (Teamwork/Task Management, Q4–Q11) and Factor 2 (Leadership/Communication, Q1–Q3). The interfactor correlation was 0.908 (95% CI 0.902–0.925). CFA on the independent Subsample 2 (*n* = 666) confirmed the two-factor structure with excellent model fit (Table 4). The LOSEFER-corrected chi-square was non-significant (χ²(43) = 42.25, *p* = .506), the interfactor correlation was 0.904 (95% CI 0.884–0.935), and factor score reliability was satisfactory (ORION: F1 = 0.860, F2 = 0.880).

Both the one-factor and two-factor models demonstrated excellent fit to the data. The one-factor solution provides the most parsimonious account and aligns with the dimensionality recommended by parallel analysis. The two-factor solution, confirmed on an independent subsample, identifies a distinguishable, though highly correlated, Leadership/Communication facet (Q1–Q3). Factor loadings and fit indices for both models are presented in Table 4.

**Table 4:** Factor loadings and model fit of the s-TEAM: one-factor and two-factor solutions

Item	1-Factor	1-Factor	2-Factor CFA	
	EFA	CFA ^a^	F1 Team/TM	F2 Lead/Comm
Leadership / Communication				
1. Leadership direction	0.633	0.582	—	0.812
2. Global perspective	0.705	0.659	—	0.544
3. Effective communication	0.749	0.743	—	0.870
Teamwork				
4. Worked together	0.795	0.791	0.586	—
5. Composure and control	0.819	0.812	0.447	—
6. Positive morale	0.827	0.822	0.618	—
7. Adapted to changes	0.813	0.826	1.316	—
8. Monitored and reassessed	0.786	0.798	0.829	—
9. Anticipated potential actions	0.802	0.801	0.833	—
Task Management				
10. Prioritised tasks	0.858	0.855	0.378	—
11. Followed standards	0.802	0.785	0.446	—
**Model fit**				
Sample	S1 (*n* = 729)	S1 (*n* = 729)	S2 (*n* = 666)	
RMSEA (95% CI)	0.012 (0.012–0.013)	0.000 (0.000–0.002)	0.000 (0.000–0.001)	
CFI (95% CI)	0.999 (0.998–0.999)	0.999 (0.999–1.000)	0.999 (0.999–0.999)	
NNFI (95% CI)	1.000 (0.999–1.000)	1.001 (0.999–1.001)	1.000 (0.999–1.000)	
χ^²^/df	1.107	0.828	0.983	
LOSEFER χ^²^ (df, p)	48.70 (44, 0.289)	36.44 (44, 0.784)	42.25 (43, 0.506)	
Interfactor r (95% CI)	—	—	0.904 (0.884–0.935)	
EAP reliability	0.950	0.948	F1 = 0.860; F2 = 0.880 ^b^	

Note. EFA = exploratory factor analysis; CFA = confirmatory factor analysis; S1 = Sample 1; S2 = Subsample 2. The one-factor model was assessed via EFA (RULS extraction) on Sample 1 and a supplementary Mild-Restricted CFA on the same sample. The two-factor model was confirmed via Mild-Restricted CFA on the independent Subsample 2. Polychoric correlations with LOSEFER-corrected chi-square throughout. Em dashes (—) = loadings fixed to zero or not applicable. F1 = Teamwork/Task Management; F2 = Leadership/Communication. Loadings > 1.0 are permissible in oblique solutions based on polychoric correlations. ^a^ Supplementary CFA conducted on the same Sample 1 (*n* = 729). ^b^ ORION factor score reliability. Analyses conducted using FACTOR 12.06.07

### Comparisons across rater perspectives

s-TEAM scores differed significantly across the four rater roles (Table 5). Significant effects were found for total score (Welch’s F(3, 461.8) = 11.97, *p* < .001, η²*p* = .032), Teamwork, Task Management, and GRS (all *p* < .001), but not for Leadership (F(3, 1410) = 2.59, *p* = .051), although the p value was close to the conventional threshold for statistical significance.

Instructor-cardiologists scored significantly lower than all three other groups on total score (d = − 0.42, *p* < .001), with differences concentrated in Teamwork (d = − 0.43) and Task Management (d = − 0.40). By contrast, instructor non-cardiologists did not differ significantly from either resident group on any variable.

Peer-assessment and self-assessment scores were closely aligned, with no significant differences in total score, domain scores, or GRS (all |d| ≤ 0.09, all *p* > .15). Both resident groups scored higher than the two instructor groups combined on total score (peer vs. instructors: d = 0.32; self vs. instructors: d = 0.29; both *p* < .001), with differences concentrated in Teamwork and Task Management; Leadership ratings were comparable across all perspectives (d = 0.04, *p* = .82).

Non-parametric sensitivity analyses (Kruskal–Wallis, Mann–Whitney U) yielded concordant results throughout.

**Table 5:** s-TEAM scores by rater role: domain and total scores (*N* = 1414)

	Instructors		Residents			
	**Cardiol.** (*n* = 192)	**Non-card.** (*n* = 158)	**Peer** (*n* = 522)	**Self** (*n* = 542)	F	*p*
Leadership (Q1–Q2)	5.48 (1.86)	5.94 (1.72)	5.76 (1.91)	5.88 (1.91)	2.59	0.051
Teamwork (Q3–Q9)	20.09 (5.06)	22.21 (4.74)	22.84 (3.76)	22.74 (4.46)	16.56ᵂ	< 0.001
Task Mgmt (Q10–Q11)	5.72 (1.65)	6.35 (1.51)	6.36 (1.32)	6.25 (1.45)	7.99ᵂ	< 0.001
**Total (Q1–Q11)**	**31.29 (7.91)**	**34.50 (7.29)**	**34.95 (6.11)**	**34.87 (6.89)**	**11.97ᵂ**	**< 0.001**
GRS (Q12)	7.56 (1.53)	7.94 (1.48)	8.18 (1.21)	8.06 (1.44)	8.97ᵂ	< 0.001

Note. Values are M (SD). Leadership = items 1–2 (range 0–8); Teamwork = items 3–9 (range 0–28); Task Management = items 10–11 (range 0–8); Total = items 1–11 (range 0–44); GRS = item 12 (range 1–10). F = one-way ANOVA; ᵂ = Welch’s F (Levene’s test indicated unequal variances). Post-hoc: Games-Howell. Instructor-cardiologists scored significantly lower than all other groups on total score and all domains except Leadership (all *p* < .001). GRS computed pairwise (*n* = 1397)

### Progression across scenarios

s-TEAM total scores increased progressively across the eight scenarios, from 31.68 (SD = 7.43) in scenario 1 to 37.41 (SD = 6.12) in scenario 8 (Pearson *r* = .266, *p* < .001; linear contrast t = 10.23, *p* < .001). This trend was consistent across all four rater roles (r range 0.200–0.389, all *p* < .01) and was paralleled by the GRS (*r* = .238, *p* < .001). Internal consistency remained stable throughout (α range 0.881–0.932; scenario 1: α = 0.900, 95% CI 0.874–0.921). Non-parametric sensitivity analyses (Spearman ρ = 0.271; Jonckheere–Terpstra z = 10.16; both *p* < .001) confirmed the monotonic upward trend.

## Discussion

The s-TEAM demonstrated strong psychometric properties across all indicators examined, supporting its use as a valid and reliable European Spanish adaptation of the TEAM for assessing teamwork in simulated cardiology emergencies. With 1,414 valid ratings, this is, to our knowledge, the largest analytical sample reported in a TEAM validation study to date, and the first multi-perspective TEAM validation to include peer-assessment. Internal consistency was excellent (α = 0.921), concurrent validity was supported by a strong correlation with the GRS (*r* = .816), and all items contributed meaningfully to the construct. These findings are broadly consistent with the psychometric evidence accumulated across 12 previous studies reporting TEAM psychometric data conducted in seven countries and diverse clinical and educational settings (Table 6). Beyond replicating the core measurement properties of the TEAM, the present study extends the evidence base in three directions: the simultaneous inclusion of four rater perspectives, the characterisation of the scale’s factor structure using polychoric correlations on independent subsamples, and the demonstration that reliable scores can be obtained without formal rater training.

**Table 6:** Psychometric properties of the TEAM across validation studies

Study	Country	Year	Context	Raters	*n*	α	IIr	ITr	ICC	Factors	*r* GRS
Cooper et al. [20]	Australia	2010	Sim (video)	1 expert	56 + 15	0.89–0.97	0.62–1.0	0.80–0.94	K 0.55	1 F (80%)	*p*<.01
Cooper & Cant [21]	Australia	2014	Sim + clin.	Expert/instr.	56–97	0.91–0.97	0.21–0.84	0.58–0.91	—	1–2 F	0.84–0.90
Cooper et al. [39]	Australia	2016	Clinical	Nurse obs.	106	0.94	0.38–0.79	0.64–0.88	0.94	1 F (64%)	0.898
Cant et al. [40]	Australia	2016	Clinical	RNs + MDs	283	0.78	M=0.45	0.41–0.62	0.78	—	0.62
Maignan et al. [22]	France	2016	Simulation	9 physicians	90	0.95	0.47–0.85	0.64–0.79	0.93	—	ρ = 0.94
Freytag et al. [23]	Germany	2019	Simulation	Nov.+expert	84	0.85–0.89	0.29–0.75	0.38–0.81	0.66	1 F (59–65%)	0.85–0.90
Karlgren et al. [41]	Sweden	2021	Clinical	Trained obs.	78	0.955	0.43–0.86	0.70–0.89	0.74	1 F (74%)	—
Giugni et al. [42]	Brazil	2022	Sim (video)	3 physicians	69	0.89	M=0.46	—	0.86	—	0.27–0.83
Carpini et al. [43]	Australia	2021	Sim (O&G)	Professionals	452	0.91–0.93	—	0.53–0.83	0.98	3 F (CFA)	0.62–0.81
Armijo-Rivera et al. [44]	Chile	2023	Simulation	Instr.+self	172	0.76–0.92	—	—	—	—	—
Morian et al. [45]	Sweden	2023	Sim (distr.)	3 raters	54	0.89–0.97	—	—	0.74–0.92	—	> 0.92
Wespi et al. [46]	Switz.	2024	VR sim.	2 trained	20	0.72–0.90	0.35–0.82	—	0.75–0.91	—	> 0.80
**s-TEAM**	**Spain**	**2026**	**Simulation**	**4 rater types**	**1414**	**0.92**	**0.34–0.82**	**0.60–0.76**	**—**	**1 F (PA); 2 F (CFA)**	**0.816**
				*Instr.-cardiol.*	*192*	*0.94*					
				*Instr. non-card.*	*158*	*0.94*					
				*Peer-assessment*	*522*	*0.90*					
				*Self-assessment*	*542*	*0.92*					

Note. α = Cronbach’s alpha, computed on the 11-item scale unless noted (the Swedish validation [41] computed α for the full 12-item instrument); IIr = inter-item correlation range; ITr = corrected item–total correlation range; ICC = intraclass correlation coefficient; r GRS = correlation with Global Rating Scale. K = Cohen’s kappa. PA = parallel analysis; EFA = exploratory factor analysis; CFA = confirmatory factor analysis. O&G = obstetrics and gynaecology; VR = virtual reality; Sim = simulation; distr. = distributed; Nov. = novice; obs. = observers; clin. = clinical. F = factor(s). Dash (—) = not reported. The s-TEAM row (bold) reports overall values; subgroup α shown in italic

***Factor structure and dimensionality ***Most previous TEAM studies using exploratory approaches identified a single factor accounting for 64–80% of the variance [20, 39, 41], while Carpini et al. [43] reported a hierarchical three-factor structure via CFA without specifying the use of polychoric correlations. In the present study, optimal parallel analysis based on polychoric correlations recommended retaining a single factor, and the one-factor EFA yielded excellent fit with 64.8% of variance explained, a figure virtually identical to the 64% reported by Cooper et al. [39] in clinical resuscitation teams and consistent with the 59–65% range observed in the German validation [23], although direct comparison should be made with caution given that previous studies employed principal component analysis on Pearson correlations rather than the polychoric-based EFA used here. The loading range (0.633–0.858) was also comparable to those reported in the original development study (0.64–0.88) [20]. However, inspection of standardised residuals revealed a systematic pattern of local dependence within the original TEAM domains, with the Leadership pair (Q1–Q2) exhibiting the largest residual (13.69 in CFA) and the lowest factor loadings (0.582–0.659). This empirical pattern, rather than an a priori theoretical expectation, motivated the exploration of a two-factor model, which yielded interfactor correlations of 0.908 and 0.904 in EFA and CFA, respectively. These results indicate that the s-TEAM is best characterised as a predominantly unidimensional measure of team emergency performance, within which a Leadership/Communication facet, comprising team direction (Q1), global perspective (Q2), and effective communication (Q3), can be identified as a distinguishable but not independent component. Notably, Q3 loaded on the Leadership rather than the Teamwork factor, departing from the original TEAM grouping but consistent with evidence that effective communication in emergency settings is closely linked to the team leader’s directive function [7, 47]. The high interfactor correlation is consistent with the single-factor solutions reported by most previous studies, while refining that interpretation: the scale captures a general team performance construct in which leadership constitutes an identifiable nuance. From an educational perspective, this supports the continued use of the total score as the primary metric, while recognising that leadership-related items may warrant specific attention during debriefing. This finding also resonates with Carpini et al.’s [43] recommendation to consider the sub-factors alongside the aggregate TEAM score when interpreting team performance.

***Rater perspectives: peer-assessment and self-assessment*** One of the most distinctive contributions of this study is the inclusion of peer-assessment, defined as the evaluation of a team’s performance by residents observing from outside the scenario, as a distinct rater perspective within the TEAM framework. To our knowledge, no previous TEAM validation study has examined this modality. Freytag et al. [23] compared novice and expert raters, but all were external observers; Armijo-Rivera et al. [44] explored self-assessment by undergraduate medical students but did not implement peer-assessment in the sense used here. In the present study, peer-assessors produced internally consistent scores (α = 0.901) and their ratings did not differ significantly from those of self-assessors in total score, domain scores, or GRS (all |d| ≤ 0.09, all *p* > .15). Self-assessment by residents who had actively participated in the scenario also yielded excellent reliability (α = 0.918). The convergence between peer- and self-assessment, and the absence of significant differences between resident and non-cardiologist instructor ratings, were consistent with our a priori hypothesis that resident-generated ratings would align with instructor ratings; the exception was the systematically lower scoring of instructor-cardiologists, discussed below. This pattern is noteworthy because peer-assessment has well-documented limitations in other educational contexts, including potential leniency bias and variability in rater standards [48], and because the general self-assessment literature reports that individuals tend to overestimate their own competence [49]. Two considerations qualify this interpretation. First, the s-TEAM assesses team-level performance rather than individual skills, which may attenuate self-serving bias. Second, residents and non-cardiologist instructors alike scored higher than instructor-cardiologists, so a degree of leniency shared by all raters without cardiology-specific expertise cannot be excluded. The fact that residents, postgraduates with clinical experience but naïve to the TEAM instrument, could produce reliable and convergent ratings has practical implications: it enables multi-source feedback in simulation training without requiring additional faculty, increasing the volume and diversity of evaluative information available for debriefing, and, if confirmed in other settings, could extend structured teamwork assessment to every participant in a simulation activity. Armijo-Rivera et al. [44] reported a similar convergence between instructors and undergraduate students using the Chilean Spanish TEAM, but in a pre-graduate population; the present findings extend this evidence to postgraduate trainees with greater clinical exposure and arguably more capacity for reflective practice.

***Instructor specialty and scoring stringency*** An unexpected finding was that instructor-cardiologists scored team performance significantly lower than the three other rater groups in Teamwork (d = − 0.43), Task Management (d = − 0.40), and total score (d = − 0.42, *p* < .001), whereas Leadership ratings did not differ significantly across perspectives (*p* = .051), suggesting that the assessment of team direction is relatively consistent across rater perspectives. Two complementary interpretations deserve consideration. First, specialty-specific expertise may increase stringency: cardiologists, sharing the same specialty as the residents, may have evaluated Teamwork and Task Management against their own discipline-specific standards, whereas non-cardiologist instructors, despite comparable simulation experience, may have applied more generic criteria. Cant et al. [40] described a related self-referential mechanism, although in their study it manifested in the opposite direction, as medical staff rating Leadership more favourably than nurses did; in our data, by contrast, Leadership was the only domain in which all perspectives converged. Second, and conversely, the same pattern can be read as a degree of leniency shared by all raters without cardiology-specific expertise (non-cardiologist instructors, peers, and self-assessors alike), with only the specialty experts applying stricter standards; in the absence of an external criterion measure, our design cannot distinguish between expert stringency and non-expert leniency. Whatever the mechanism, this observation has practical implications for rater calibration in simulation programmes: when multiple instructor perspectives are used, awareness of potential specialty-related stringency differences is important, and averaging across perspectives may provide a more balanced assessment.

***Feasibility without prior training and performance progression*** Internal consistency was excellent from the first scenario (α = 0.900, 95% CI 0.874–0.921) and remained stable throughout the eight scenarios (range 0.881–0.932), even though neither instructors nor residents had used the TEAM instrument before the study. Cooper et al. [20, 39] reported that the TEAM was feasible with minimal training, and Freytag et al. [23] demonstrated comparable ratings between novice and expert raters after a structured rater training. The present study goes further: reliable scores were obtained without formal rater training or calibration, with instrument-specific preparation limited to a brief familiarisation (an explanation of approximately 10 min for instructors and of approximately 5 min for residents). This finding should be interpreted against the recommendation to formally train raters for structured team-performance assessment [50]. Several features of our setting may explain why internally consistent ratings were nonetheless achieved: the TEAM items are short and behaviourally anchored, and the instrument was designed for rapid observational use [20, 21]; instructors were familiar with team-performance concepts from previous simulation-based training, and residents, although they had received no theoretical instruction in non-technical skills and their prior knowledge was heterogeneous, were clinicians-in-training with daily exposure to teamwork in acute cardiovascular care; and ratings referred to team-level rather than individual performance, arguably a less inferentially demanding judgement. We do not interpret these results as evidence that rater training is unnecessary: internal consistency does not guarantee calibration, and the specialty-related differences in stringency described above are precisely the type of effect that formal training might mitigate. Rather, our findings suggest that, when formal rater training is not feasible in large-scale programmes, the s-TEAM can still yield internally consistent scores. Meanwhile, total scores increased progressively across scenarios (Pearson *r* = .266, *p* < .001) while measurement quality remained stable, a pattern compatible with genuine gains in team performance; nevertheless, in the absence of objective clinical performance data, this interpretation remains tentative (see Limitations). This combination of stable measurement and improving scores supports the use of the s-TEAM as both an assessment tool and a formative instrument within iterative simulation curricula.

### Limitations

Several limitations should be acknowledged. First, the study was conducted in a single simulation centre using cardiology emergency scenarios, which may limit generalisability to other specialties and clinical settings. Second, the fully anonymous data collection, while protecting participant confidentiality, precluded multilevel modelling of rater-level variance and the computation of formal inter-rater reliability coefficients (ICC) between individual rater pairs. Although ICC has been reported in most previous TEAM validation studies (range 0.55–0.98 across studies; Table 6), the anonymous design, in which only the rater category (instructor-cardiologist, instructor non-cardiologist, peer, self) was recorded without individual identifiers, made it impossible to link repeated ratings to specific individuals, a prerequisite for ICC computation. Nevertheless, the convergence of scores between peer-assessment and self-assessment (|d| ≤ 0.09) and the consistently high internal consistency across all four rater groups provide indirect evidence of inter-perspective agreement. Third, test–retest reliability was not assessed. Fourth, ceiling effects were observed on several Teamwork items (up to 55.9% for Q6), which may attenuate variance and inflate inter-item correlations; this pattern has been reported in previous TEAM studies [22, 41] and may reflect the high overall performance of trained teams in simulation settings. Fifth, the 1,414 observations are nested within teams and course editions, potentially violating the independence assumption of the ANOVA and factor analyses; the anonymous design precluded multilevel modelling to account for this clustering. Sixth, the absence of demographic data (sex, age, prior experience) precluded measurement invariance analyses and limits the characterisation of the sample’s representativeness. Finally, the absence of an external criterion measure (e.g., clinical outcomes or Observational Skill-based Clinical Assessment tool for Resuscitation (OSCAR) scores [51]) limits the assessment of criterion validity to the internal GRS. For the same reason, the progressive increase in scores across scenarios, although consistent across all four rater perspectives, is not corroborated by objective performance data (e.g., faster or more effective clinical management) and may be partly influenced by the shared expectation of participants and instructors that performance improves over a simulation course.

## Conclusions

The s-TEAM is a valid, reliable, and feasible European Spanish adaptation of the TEAM for assessing non-technical teamwork skills in simulated cardiology emergencies. Its psychometric properties are consistent with the international body of evidence accumulated over 15 years of TEAM research. Parallel analysis recommended a single factor, and both one-factor and two-factor models demonstrated excellent fit, supporting a predominantly unidimensional structure with a distinguishable Leadership/Communication facet. Importantly, reliable scores were obtained across four distinct rater perspectives, including peer-assessment, examined here, to our knowledge, for the first time within the TEAM framework, and without formal rater training. These findings support the integration of the s-TEAM into simulation-based training programmes as a tool for multi-source assessment, with potential to enhance both individual and team learning in emergency healthcare education.

## Supplementary Information

Below is the link to the electronic supplementary material.

Supplementary Material 1

Supplementary Material 2

## Data Availability

The datasets used and/or analysed during the current study are available from the corresponding author on reasonable request.

## Abbreviations

ANOVA: Analysis of variance
CFA: Confirmatory factor analysis
CFI: Comparative fit index
CI: Confidence interval
EAP: Expected a posteriori
EFA: Exploratory factor analysis
GRS: Global rating scale
ICC: Intraclass correlation coefficient
KMO: Kaiser–Meyer–Olkin
NNFI: Non-normed fit index
OSCAR: Observational skill-based clinical assessment tool for resuscitation
PEARLS: Promoting excellence and reflective learning in simulation
RMSEA: Root mean square error of approximation
RULS: Robust unweighted least squares
SD: Standard deviation
SEC: Sociedad Española de Cardiología (Spanish Society of Cardiology)
s-TEAM: European Spanish version of the team emergency assessment measure
TEAM: Team emergency assessment measure
