# Supplementary material for: Translation, cross-cultural adaptation, and psychometric validation of the Team Emergency Assessment Measure (TEAM) into European Spanish: a multi-perspective study including peer-assessment
Source: Adv Simul (Lond). 2026 Sep 9;11:61. doi: 10.1186/s41077-026-00484-1 (PMC13579855; doi:10.1186/s41077-026-00484-1)
Supplement: Supplementary file 1 [file 41077_2026_484_MOESM1_ESM.docx]

**Additional file 1: Participant exclusion flowchart.**

**Additional file 1**

Participant exclusion flowchart

s-TEAM forms collected
N = 1,541
(9 course editions × 3 teams × 8 scenarios × ~4 raters)

↓

*Excluded prior to data entry: n = 43
(illegible or entirely blank)*

Digitised records
n = 1,498

↓

*Excluded for missing item responses (Q1–Q11): n = 82
Excluded for out-of-range values: n = 2*

Final analytical sample (complete Q1–Q11)
N = 1,414

↓

| **Rater perspective** | **n** |
| --- | --- |
| Instructor-cardiologists | 192 |
| Instructor non-cardiologists | 158 |
| Residents — peer-assessment (observers) | 522 |
| Residents — self-assessment (performers) | 542 |

↓

*Forms without scenario identification: n = 42
(retained in main analyses; excluded from scenario-level analyses only)*

Scenario-level subsample
n = 1,372 (97.0%)

**Note:** No imputation was performed. Listwise deletion was applied for all analyses involving the 11-item scale (Q1–Q11). The 17 forms with missing Global Rating Scale (item 12) values were handled via pairwise deletion (n = 1,397 for GRS-related analyses). Data collection was fully anonymous; no demographic data were recorded.
